# Optimizing Female Warfighter Health and Performance in Environmental Extremes

**Published:** 2025-05-01

**Authors:** Gabrielle E. W. Giersch, Karleigh E. Bradbury, Nisha Charkoudian

**Affiliations:** Thermal and Mountain Medicine Division, U.S. Army Research Institute of Environmental Medicine, Natick, MA

The ongoing non-combat threats of extreme environmental exposure—including heat, cold, high altitudes, and subterranean areas—have always been important focuses for research and development of effective countermeasures, both within the military as well as other domains requiring prolonged outdoor activity. Over the past decade, the increased frequency of weather phenomena associated with climate change—such as extreme heat waves, violent storms, catastrophic flooding—have brought the biomedical risks and gaps in knowledge associated with extreme environmental exposure into sharper focus, both in the biomedical literature and mainstream media. ^1 , 2^

In military medicine, threats due to environmental exposure are particularly relevant, as small units and dismounted warfighters are often directly exposed to a given set of extreme environmental conditions. Until the past decade, much of the military medical literature on environmental stressors relied on data collected primarily from men. With women representing a growing percentage of the military force, both in the U.S. and around the world, it is increasingly necessary to understand the physiological and pathophysiological responses of both sexes for optimizing the health of all warfighters, in mission-critical scenarios as well as training.

The environmental physiology community, including the U.S. Army Research Institute of Environmental Medicine (USARIEM) and many academic laboratories and research teams, have recently increased focus on the female warfighter, to improve knowledge and awareness of individualized risk management for individuals exposed to extreme terrestrial environments. ^3 - 8^ This editorial provides an update on the state of the science to aid military medical providers' understanding of where and when sex-specific considerations in environmental exposure risk are necessary and—equally importantly—are not.

## Threats of Extreme Environmental Exposure

### Heat Stress

Heat-related illnesses remain a major threat to warfighters, with 2,000 or more individuals each year experiencing mild forms of heat illness, and approximately 500 individuals experiencing exertional heat stroke (EHS), which is the most severe, and potentially fatal, heat illness. It was previously assumed that women are at an increased risk for heat-related illnesses compared to their male counterparts, ^9 , 10^ without appropriate evidence.

Recently, the Thermal and Mountain Medicine Division of USARIEM evaluated epidemiological data, using a case-control analysis, from an Army-wide analysis of EHS, the most severe form of heat illness, and found no differences in risk of EHS between men and women. ^11 , 12^ While sex-related differences for EHS risk are not apparent, recent evidence suggests potential differences in organ damage between men and women following EHS. In a cohort of EHS cases from Fort Benning, the Thermal and Mountain Medicine Division of USARIEM also found that biomarkers of end organ damage were lower in women compared to men, despite similar highest recorded body temperatures. ^13^ This may suggest that women suffer less damage than men from EHS of similar severity, but more research is needed to fully elucidate physiological mechanisms to support that hypothesis.

Risk for developing other less severe, heat-related illnesses has recently been evaluated. Kazman et al. (2024) reported an increased risk for “mild” heat illness (i.e., heat exhaustion) in recruits, ^14^ but more research to elucidate the mechanism for this potential difference is warranted. In the yearly heat illness active updates from *MSMR* , men and women consistently demonstrate similar rates of reported heat exhaustion, including heat injury (diagnosed as heat exhaustion with evidence of end organ damage).

While risk of heat-related illness does not appear to be affected by sex, relatively low-impact differences in thermoregulation between men and women exist. Namely, and likely the most effective, are the physical differences between men and women, where women are, on average, smaller with larger body surface area to mass ration (BSA:mass ^-1^ ), which can enhance heat dissipation in some environments. ^15^ This may provide women with a relative advantage in certain situations where heat dissipation is not impeded by clothing or the surrounding environment. Situations in which heat dissipation capacity is impeded, or work rate is very high (i.e., load carriage occurs), instead could turn women's BSA:mass ^-1^ into a disadvantage; lower lean body mass carrying an absolute load in clothing with less evaporative capacity leads to greater thermal stress. Additionally, there is evidence to suggest lower sweating rate in women at very high work loads, ^16 , 17^ which may affect military personnel when performing ruck marches or runs, but also may not yield significant differences in total heat loss or core temperature responses, despite less sweat produced. ^18^

Female sex hormones also have pronounced effects on the thermoregulatory system, as previously thoroughly described, ^19^ but those hormonal or menstrual cycle differences do not seem to affect body temperature following exercise, when evaluated in a meta-analysis. ^20^ Contraceptive status did appear to influence esophageal temperature during exercise, ^21^ with progestin-only contraceptive users experiencing higher core temperatures at baseline and through-out exercise, which may suggest that type of contraceptive influences thermoregulatory response in women.

Besides physiological differences, there are also well-described behavioral differences in thermoregulation between men and women, including pacing ^22^ and behavioral thermoregulation. ^23^ Behavioral mechanisms may be more effective than physiological mechanisms for thermoregulation ^24^ and should be considered for future research in addition to practical prevention strategies.

### Cold Stress

Female-specific responses during cold stress are less well-understood. Female sex hormones and cold stress responses have been thoroughly reviewed, ^7^ with findings indicating that estradiol may enhance cutaneous vasodilatory responses ^25 - 27^ and progesterone observed to shift the core temperature threshold to higher temperatures, ^25 , 28^ which may affect shivering onset and sensitivity ^29 , 30^ (although that finding varies in the literature, ^31^ without consistent methodologies in reported investigations). Interestingly, if elevated progesterone concentrations, like those observed in the luteal phase of the menstrual cycle, do influence shivering onset, it is likely such effect is due to elevated core temperatures observed with high progesterone and would not influence risk of injury, impaired performance, nor loss of dexterity during cold stress.

Manual dexterity decreases with cold stress, and cold-induced vasodilation, a reflex that opens the blood vessels to the extremities to provide blood flow, likely occurs to prevent freezing cold injuries. ^32^ Women have been shown to have lower finger temperatures than men, with no differences in cold-induced vasodilation or dexterity measures between sexes, ^33^ but sex hormones were not evaluated systematically in that investigation.

The impacts of estradiol and progesterone, particularly with fluctuations that occur not only during the menstrual cycle, but throughout reproductive lifespan as well as with contraceptive use, require further investigation.

Women do appear to have a greater prevalence of Raynaud's phenomenon, ^34 , 35^ but there is currently not evidence to suggest specific influence of female sex hormones or menstrual cycle phases on Raynaud's prevalence or severity. ^36^ Raynaud's phenomenon may pose an increased risk in developing freezing cold injuries such as frostbite. ^37^

The physical differences that largely govern differences between men and women in heat stress may also affect cold stress, with greater subcutaneous fat potentially providing insulation in women ^38^ and greater average BSA:mass ^-1^ potentially increasing heat loss in cold environments. It is unclear if these physical differences influence risk of injury or decreased performance during cold stress.

### High Altitude Stress

It is well-established that humans experience myriad physiological responses upon exposure to high terrestrial altitude due to associated decrease in partial pressure of oxygen (PO _2_ ). ^39^ The decrease in PO _2_ is the primary cause of the decrements in both physical and cognitive performance ^40^ that occur with altitude exposure.

Both acute and chronic responses to hypoxia occur with a primary goal of increasing oxygen delivery throughout the body. Acute responses include, but are not limited to, increases in heart rate, ventilation, diuresis, and sympathetic nervous system activity. ^3 , 41^ Longer-term adaptations, known as acclimatization, include increases in hemoglobin mass, alterations in substrate utilization (e.g., enhanced carbohydrate metabolism), and improvements in capillary O _2_ extraction. ^40^ The current literature presents some sex differences in physiological variables such as ventilation and hemoglobin mass at sea level baseline, but these differences do not appear to influence acute physiological responses to altitude nor the process of acclimatization in women. ^41^

Ventilation has been shown to be influenced by sex and female sex hormones. ^42^ Progesterone is a ventilatory stimulant that acts on both the peripheral and central chemoreceptors to increase ventilation ^41^ and, concomitantly, lower partial pressures of end tidal CO _2_ (PETCO _2_ ). Higher ventilation and lower PETCO _2_ have been reported in women in comparison to men, as well as during the luteal versus follicular phase of the menstrual cycle. ^43^ Despite the presence of progesterone, there are no sex differences in the acute hypoxic ventilatory response ^43^ or in the time course of ventilatory acclimatization. ^44^

One potential limitation for females at altitude is an increased work of breathing. Women experience increased work of breathing compared to men, even when corrected for body and lung size, ^45^ a result of smaller airways that increase air flow resistance during ventilation. ^46^ This physiological difference between sexes may be of particular importance during load carriage at altitude, when increased work of breathing intensifies due to increased thoracic resistance caused by the external load.

Increased work of breathing for a specific ventilation is caused by elevated oxygen demand and greater respiratory muscle use. ^47 , 48^ In situations when respiratory muscle metabolic rates are increased, blood flow is redistributed to the diaphragm and other respiratory muscles because of increased sympathetic nerve activity, concomitant with vasoconstriction to blood vessels of other active or inactive muscles (i.e., respiratory muscle metaboreflex). Recent work has demonstrated attenuated sympathetic responses in women to increases in inspiratory muscle work, both at rest ^49^ and during exercise, ^50^ but the implications of these findings for blood flow redistribution during high levels of respiratory muscle work (i.e., during load carriage at altitude), and whether women are at a disadvantage due to increased work of breathing, are unknown. ^51 , 52^

High altitude illness is an important risk consideration for those ascending in altitude. The most common high altitude illness is acute mountain sickness (AMS), which occurs when individuals rapidly ascend to altitudes to 2,500 meters or higher, with symptom prevalence and severity increasing with higher elevations. ^53 , 54^ Symptoms of AMS often include nausea, dizziness, fatigue, headache, and gastrointestinal distress. Other, but less common, altitude-associated illnesses include high altitude cerebral and pulmonary edemas (HACE and HAPE, respectively). One formative study reported that men may be more likely to develop AMS, ^55^ but other reports suggest that women may be at a greater risk ^56^ —or that there are no differences between the sexes. ^53^ Additional research to investigate potential sex differences in high altitude illness risk, while also exploring potential mechanisms, are needed.

### Subterranean and Hypercapnic Environments

Environments with elevated levels of carbon dioxide, which can result in hypercapnia, are an environmental stressor that recently has gained interest in the military community, after relatively little research. In normal ambient air, the concentration of carbon dioxide (CO _2_ ) is very low (~0.034%), but in certain situations such as subterranean environments (including underground tunnels and buildings), ^57^ or when individuals are wearing personal protective equipment, ^58^ CO _2_ concentration can be as high as 7%. ^59^ Small increases in ambient CO _2_ can cause symptoms such as decreased focus, fatigue, and weakness, as well as increased levels of anxiety, but higher concentrations (10-15%) can lead to unconsciousness and suffocation within minutes of exposure, with further increases resulting in death. ^60^

Similar to hypoxia, hypercapnia triggers a robust ventilatory response. ^61 , 62^ Several investigations have sought to distinguish sex differences during ventilatory response to increased CO _2_ , known as hypercapnic ventilatory response (HCVR). Some investigations have reported a greater HCVR in men, but when corrected for body size—including body mass index, body surface area, and lung size—those differences no longer exist. ^63 , 64^ While HCVR appears to be similar in both sexes, the ventilatory threshold for increase in ventilation is lower in women. ^65^

Previous studies have explored the influence of increased CO _2_ on both physical ^59^ and cognitive performance, ^66^ but those studies included few women. Sex differences in physiological responses to increased CO _2_ remains to be investigated.

## Editorial Comment

With the increased proportional and absolute numbers of women in the U.S. military, it is vital to better understand female physiological responses to, and unique requirements for, environmental extremes. Continued research should seek to investigate these potential sex differences to ensure suitable policy recommendations for both women and men in uniform.
